# Feasibility, Reproducibility, and Agreement between Different Speckle Tracking Echocardiographic Techniques for the Assessment of Longitudinal Deformation

**DOI:** 10.1155/2013/297895

**Published:** 2013-09-22

**Authors:** Sergio Buccheri, Ines Monte, Sarah Mangiafico, Vera Bottari, Stefano Leggio, Corrado Tamburino

**Affiliations:** ^1^Department of Medical and Pediatric Sciences, University of Catania, Via Plebiscito 628, 95124 Catania, Italy; ^2^Clinical Echocardiography, A.U.O. Policlinic “Vittorio Emanuele”, P.O. Rodolico, Via Santa Sofia 78, 95125 Catania, Italy; ^3^Cardiology Unit, Ferrarotto Hospital, Via S. Citelli 6, 95124 Catania, Italy

## Abstract

*Background.* Left ventricular (LV) longitudinal deformation can be assessed with new echocardiographic techniques like triplane echocardiography (3PE) and four-dimensional echocardiography (4DE). We aimed to assess the feasibility, reproducibility, and agreement between these different speckle-tracking techniques for the assessment of longitudinal deformation. *Methods*. 101 consecutive subjects underwent echocardiographic examination. 2D cine loops from the apical views, a triplane view, and an LV 4D full volume were acquired in all subjects. LV longitudinal strain was obtained for each imaging modality. *Results*. 2DE analysis of LV strain was feasible in 90/101 subjects, 3PE strain in 89/101, and 4DE strain in 90/101. The mean value of 2DE and 3PE longitudinal strains was significantly higher with respect to 4DE. The relationship between 2DE and 3PE derived strains (*r* = 0.782) was significantly higher (*z* = 3.72, *P* < 0.001) than that between 2DE and 4DE (*r* = 0.429) and that between 3PE and 4DE (*r* = 0.510; *z* = 3.09, *P* = 0.001). The mean bias between 2DE and 4DE strains was −6.61 ± 7.31% while −6.42 ± 6.81% between 3PE and 4DE strains; the bias between 2DE and 3PE strain was of 0.21 ± 4.16%. Intraobserver and interobserver variabilities were acceptable among the techniques. *Conclusions*. Echocardiographic techniques for the assessment of longitudinal deformation are not interchangeable, and further studies are needed to assess specific reference values.

## 1. Introduction

Recent advances in the field of speckle tracking echocardiography (STE) have allowed the assessment of myocardial deformation. Speckles are natural acoustic markers, determined by interference patterns within an ultrasonic window that can be identified by dedicated softwares and followed during the entire cardiac cycle. The frame-to-frame tracking allows the assessment of angle independent two-dimensional (2D) and three-dimensional (3D) sequences of tissue motion and deformation [1] obtaining new parameters of cardiac function like strain. Strain represents a dimensionless index that expresses the deformation of a myocardial segment during the cardiac cycle in respect to its initial length [2].

The angle independency of this technique allows the assessment of myocardial deformation along the longitudinal, radial, and circumferential spatial directions. Longitudinal function is generally the most sensitive to myocardial injuries, and longitudinal strain has in fact shown interesting potentialities for the early identification of myocardial dysfunction, often before impairment of conventional echocardiographic parameters like ejection fraction [3, 4]. 

Longitudinal deformation, conventionally determined by 2D echocardiography (2DE), can nowadays be assessed with the use of new echocardiographic real time multidimensional techniques like triplane echocardiography (3PE) and real time four-dimensional echocardiography (4DE). Few studies have been performed to assess the feasibility of these new techniques and the consistency of deformational measurements among the different methods. Accordingly, we aimed to assess the feasibility, reproducibility, and agreement between these different STE techniques for the assessment of longitudinal deformation.

## 2. Methods

### 2.1. Study Population

This was a single centre and prospective study. From December 2012 to February 2013, all consecutive subjects referred to our echo lab for an echocardiographic examination were prospectively enrolled. A group of healthy volunteers was also included in the study. Exclusion criteria were represented by the presence of supraventricular arrhythmias (atrial fibrillation or flutter). No other specific exclusions' criteria were adopted. 

The final study population was represented by a total of 101 subjects. Reasons for referral were represented by echocardiographic examination after patent foramen ovale (PFO) and atrial septal defect (ASD) percutaneous closure, systemic arterial hypertension, presence of cardiac murmurs, and routine echocardiographic examination in athletes.  Table 1 shows the clinical characteristics of the study population (see Table 1). 

All subjects were informed before the echocardiographic examination of the study's purposes. A written informed consent was achieved from all the subjects for the inclusion in the study. 

### 2.2. Echocardiographic Examination

All subjects underwent echocardiographic examination by using a GE Vivid 9 equipped with an MS5 probe and a matrix array 4V probe. The echo protocol included the initial acquisition of 2DE cine loops from the apical 4 chamber, 2 chamber, and apical long-axis views. Transducer's position was optimized to avoid foreshortening during cine loops' acquisition. The optimal frame rate, not precluding the clear visualization of myocardial boundaries, was achieved. Three consecutive heart cycles were stored.

After 2DE acquisition, the 4V matrix array probe was initially used to obtain 3PE cine loops. 3PE allows the simultaneous evaluation of the three apical views in the same cardiac cycle. For the triplanar acquisition, the same apical position was used. The frame rate was optimized also in this case with acquisition of three heart cycles.

For the acquisition of 4DE cine loops, the multislice modality (12 slices) was initially selected. This allowed the assessment of 9 sequential ventricular short-axis views (from the base to the apex) together with the apical 4, 2 chambers and apical long-axis views. Careful attention was paid to ensure that all ventricular segments, including the apex, were included in the echo image. Then, the number of cardiac cycles to be included in the multibeat acquisition was set from 2 to 6 heartbeats. The multi-beat acquisition allowed the sequential reconstruction of wedge-shaped subvolumes of the left ventricle that is equivalent to the number of heart cycles initially selected. Subjects were required to breath-hold during the multibeat acquisition. All the 4DE acquisitions were performed according to the EAE/ASE recommendations for image acquisition and display using three-dimensional echocardiography [5]. Cine loops were digitally stored and transferred to a PC workstation for subsequent off-analysis. 

**Figure 1:**
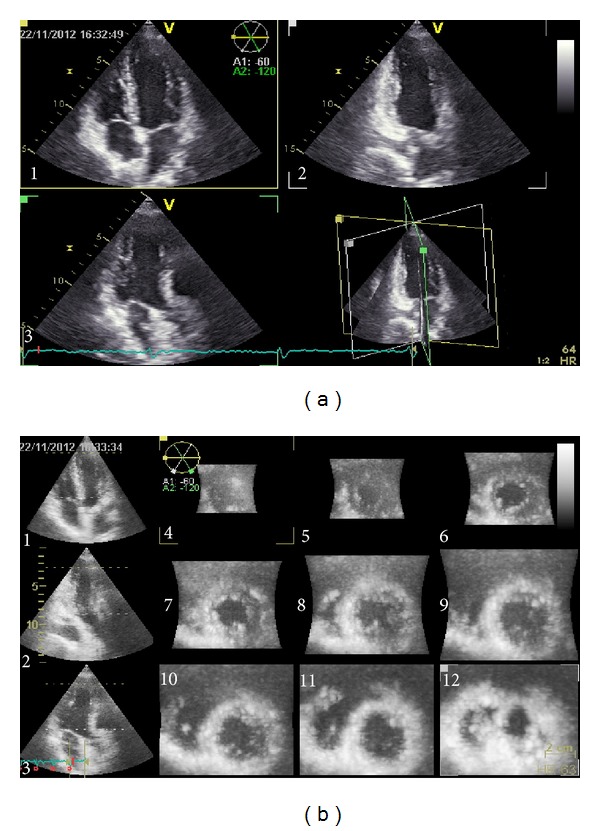
Picture (a) showing a triplane acquisition of the LV (numbers 1, 2, and 3, resp., represent the apical 4 chamber, 2 chamber, and apical long-axis views); picture (b) showing a 12-slice reconstruction of the LV (numbers 1, 2, and 3 as in picture (a); numbers from 4 to 12 showing a sequential reconstruction of short-axis views from the apex to the base of the LV).

Figure 1 shows a triplane and multislice acquisition of the left ventricle (Figure 1).

### 2.3. Speckle Tracking Analysis

Speckle tracking analysis was performed using a commercially available software (Echopac, GE Healthcare, ver. 112.0.0.). For the evaluation of 2D longitudinal strain (2D strain), the quantitative analysis (*Q*-analysis) modality was selected. This technique allows the evaluation of segmental and global myocardial longitudinal deformation using a 17-segment anatomic model of the LV. The operator defined the endocardial border from each acquisition plane by placing multiple anchoring points at end diastole, and then the software automatically detected the region of interest (ROI) from the endocardial to the epicardial layer. Careful attention was paid to ensure that all the myocardium was inside the ROI and to avoid pericardial inclusion. The tracking was then visually controlled and validated. The aortic valve closure timing was automatically selected by the software using strain curves profiles. Global longitudinal strain was obtained by averaging segmental values. Specific segments with missed visualization and/or poor tracking quality were considered as excluded segments (ES). When more than three segments were not analyzable, longitudinal strain was considered not feasible (NF).

To obtain longitudinal strain from the 3PE cine loops, the automated functional imaging (AFI) modality was selected. Triplanar AFI allows the separate identification of ROIs from planes included in the simultaneous 3P image. ROI definition and segments exclusion was performed according to the 2D protocol. Even in this case, longitudinal strain (3P strain) was obtained by averaging each segmental value and when three or more segments were not analyzable, the technique was considered not feasible. 

Four-dimensional longitudinal strain (4D strain) was obtained selecting the 4D Auto LVQ modality. The following preliminary steps were performed before tracking:

- automatic alignment of the full volume acquisition (in case of suboptimal alignment manual alignment was performed),
- identification of end-diastolic and end-systolic endocardial borders by placing one point at the tip of the mitral valve and one at the apex (in case of suboptimal delineation of the endocardial borders manual adjustment was performed),
- validation of the epicardial border, which was automatically displayed by the software and eventually corrected in case of insufficient delineation.

The software then performed speckle tracking analysis following speckles within a definite volume using the conservation of mass as a restriction. Longitudinal strain was obtained by averaging segmental values. The preliminary steps required for strain analysis also allowed the identification of LV end-diastolic and end-systolic volumes (LV EDV and LV ESV, resp.), ejection fraction (LV EF), mass (LV M), sphericity index (LV SpI), and cardiac output (LV CO).

For each strain technique, the time spent for a full satisfactory analysis was measured and expressed as minutes and seconds (min. and sec., resp.).

### 2.4. Reproducibility Analysis

Intra- and interobserver variabilities are expressed as coefficient of variability (COV) and intraclass correlation coefficient (ICC) with 95% confidence interval. COV was calculated as the absolute difference of paired measurements in percent of their mean. To assess intraobserver variability, the same operator, blinded to previous measurements, performed measures after two weeks in a subgroup of 20 randomly selected subjects (8 healthy subjects and 12 subjects with comorbidities potentially affecting longitudinal deformation). For the assessment of interobserver variability, a second operator blinded to previous results repeated measures in the same group of subjects.

### 2.5. Statistical Analysis

Continuous variables are presented as mean ± standard deviation (SD) while dichotomous variables as frequencies and percentage (%). Analysis of variance (ANOVA) was used to compare continuous variables, while the chi-squared test was performed to compare categorical variables. Agreement among the different speckle tracking techniques was assessed by the Bland-Altman method of agreement [6]. Correlation between strain values was derived from each technique and was assessed by either Pearson's method or Spearman ranks test, as appropriate. Relationship between difference in frame rate/volume rate and the difference in the corresponding strain value was explored with the same method. Correlation coefficients were compared after Fisher's *z*-transformation. All tests were two tailed. A *P* value < 0.05 was considered statistically significant. Statistical analysis was performed using SPSS ver. 15 (SPSS, Inc., Chicago, Illinois).

## 3. Results

Table 1 shows the clinical features and echocardiographic parameters derived from 4DE analysis in the study population. The population included in the study had a wide range of age (14–76 years) and body mass index (18.6–36.1 kg/m^2^) according to clinical characteristics of a nonselected population. We also observed for the echocardiographic parameters a wide range of LV EF, volumes, and mass.

2D analysis of LV strain was feasible in 90/101 subjects (89.1%); 3P strain was performed in 89/101 (88.1%), while 4D strain was obtained in 90/101 subjects (89.1%). A total of 1530 segments were analyzed for 2D and 4D strain, while 1513 segments were assessed for 3P strain. 

**Figure 2:**
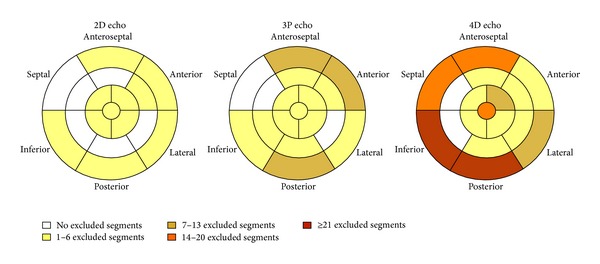
Picture showing the anatomic localization of exclusion within the 17-segment model of the LV.

The mean value of 2DE and 3PE derived longitudinal strain (−20.81 ± 3.03% and −20.82 ± 3.26%, resp.) was significantly higher in respect to 4D strain (−14.26 ± 4.02%; *F* = 106.2, *P* < 0.001). Excluded segments from analysis were 36 (2.4%) for 2DE, 54 (3.6%) for 3PE, and 155 (10.1%) for 4DE (*P* = 0.047 between 2DE and 3PE; *P* < 0.001 for 2DE and 3PE with respect to 4DE).  Figure 2 shows the localization of excluded within the 17-segment anatomic model of the LV (Figure 2). 

3PE had the minimum time required for analysis (1 min. and 26 sec. ± 25 sec.) with respect to 2DE and 4DE (1 min. and 48 sec. ± 39 sec. and 1 min. and 59 sec. ± 29 sec., resp.; *F* = 25.7,  *P* < 0.001). 

**Figure 3:**
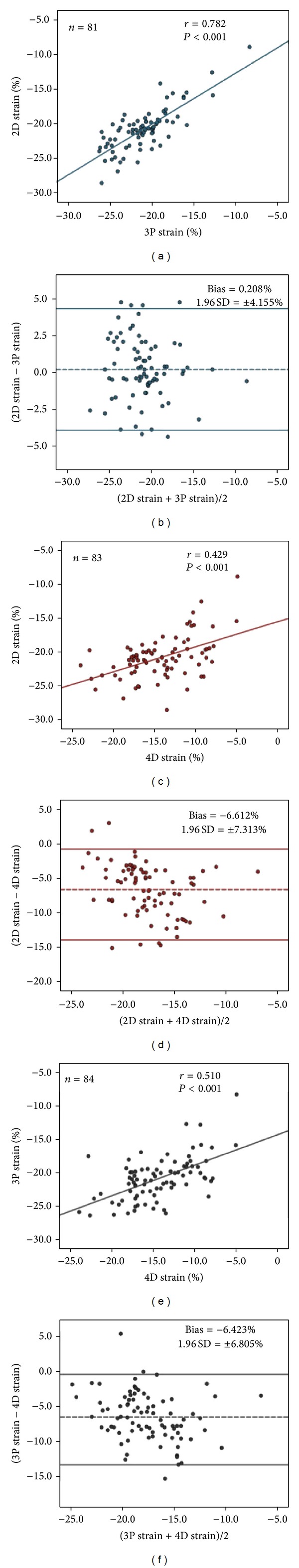
Pictures showing scatter diagrams and Bland-Altman plots among the different techniques.

The scatter diagrams and Bland-Altman plots between the different techniques are shown in Figure 3. Mean bias between 2D and 4D strains was −6.612% with limits of agreement (1.96 SD) of ±7.313%; mean bias between 3P and 4D strains was −6.423% with 1.96 SD of ±6.805%. Bias between 2D and 3P strains was of 0.208% with 1.96 SD of ±4.155%. Relationship between 2D and 3P strains was significantly higher (*z* = 3.72, *P* < 0.001) than that between 2D and 4D strains and that between 3P and 4D strains (*z* = 3.09,  *P* = 0.001). 

**Figure 4:**
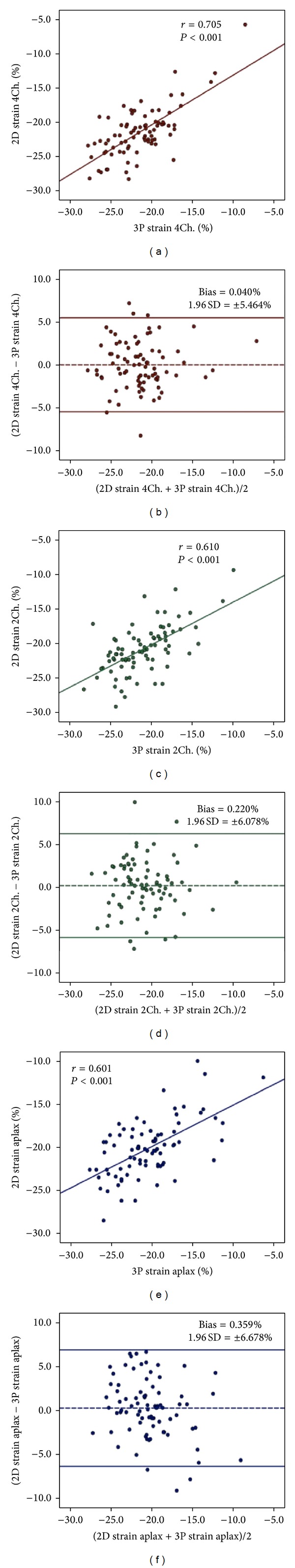
Picture showing scatter diagrams and Bland-Altman plots between apical views derived from 2DE 3P Echo.

In Figure 4, scatter diagrams and Bland-Altman plots between apical views derived from 2DE and 3PE are shown. The major source of bias was identified for the apical long-axis view (mean bias ± 1.96 SD = 0.359 ± 6.678%).

To evaluate the influence of pathological conditions upon intertechnical biases, subjects were subdivided in two groups according to the presence (PFO and ASD defect closure, arterial hypertension) or absence (athletes and healthy subjects) of comorbidities potentially influencing longitudinal strain. The overall feasibility for each technique, also including segmental feasibility, was similar between the two groups. A slight improvement in agreement between 2D and 4D strains was identified in healthy subjects in respect to subjects with pathological conditions (mean bias ± 1.96 SD of −6.240  ±6.622% and −6.927 ± 7.842%, resp.); this was also observed for the agreement between 3P and 4D strains (−6.051 ± 7.160% and −6.710 ± 6.546%, resp.); in addition, agreement between 2D and 3P strains was similar between the two groups (0.070 ± 3.902% and 0.313 ± 4.371, resp.). However, intertechnical biases did not significantly differ between the two groups (*P* = 0.406 for bias between 2DE and 4DE, *P* = 0.392 between 3PE and 4DE, and *P* = 0.612 between 2DE and 3PE). 

**Figure 5:**
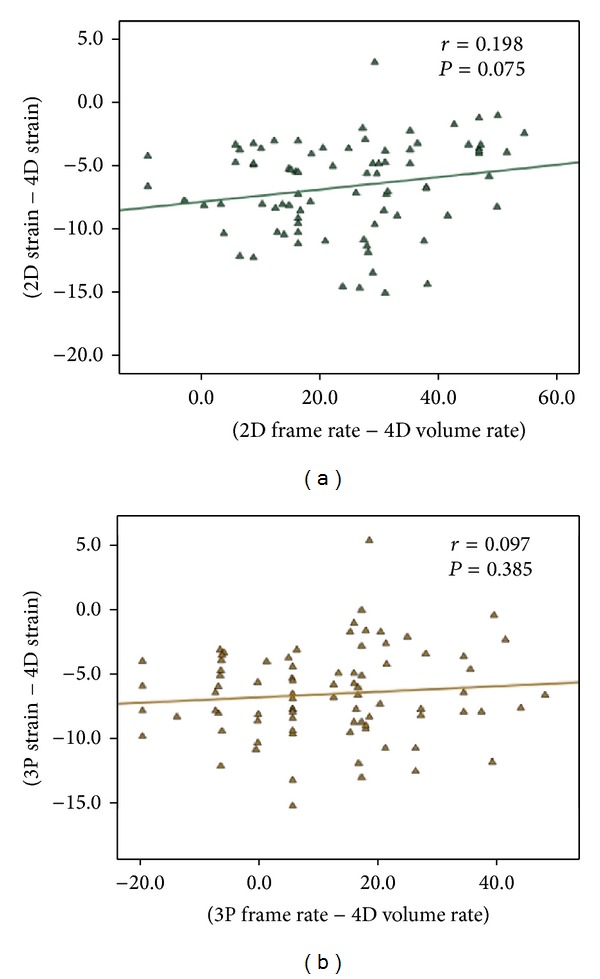
Picture showing the relationship between differences in frame rate/volume rate and the difference in the corresponding strain values.

Figure 5 shows the relationship between differences in frame rate/volume rate and the differences in the corresponding strain values. No statistically significant relationship was identified for each technique. Moreover, subjects where subdivided in two groups according to the presence of a 4DE acquisition with a volume rate > 40 volumes/sec (*n* = 30) or ≤40 volumes/sec (*n* = 58); a trend toward a significant increase in segmental feasibility was observed in subjects with a volume rate > 40 volumes/sec but this did not reach the statistical significance (112 excluded segments for subjects with ≤40 volumes/sec acquisition and 43 for subjects with >40 volumes/sec acquisition, *P* = 0.078). No improvement in agreement between 2DE and 4DE was identified in subjects with a volume rate acquisition > 40 volumes/sec in respect to subjects with a volume rate acquisition ≤ 40 volumes/sec (−6.923 ± 5.306% and −6.610 ± 7.930%, resp.; *P* = 0.769); this was also observed for bias between 3P and 4D strains (−6.492 ± 4.675% and −6.531 ± 7.605%, resp.; *P* = 0.956). 

Intra- and interobserver reproducibilities are shown in Table 2 (see Table 2). Both intra- and interobserver variabilities were acceptable among the techniques. 

## 4. Discussion

Speckle tracking echocardiography represents a promising tool for the identification of myocardial dysfunction in a preclinical setting. 2D strain actually is the most exploited technique with many clinical studies performed with the aim of identifying subclinical alterations of myocardial deformation in different cardiac diseases [7, 8]. Recent advances in the field of real time multidimensional echocardiography have allowed the assessment of cardiac deformation by means of new techniques like 3PE and 4DE. These newer approaches can overcome the weakness of 2D strain allowing a reliable analysis of myocardial deformation with new insights and potentialities for the investigation of myocardial (dys) function. 

The major findings of this study are:

- techniques actually available for the assessment of longitudinal deformation are feasible and reproducible but not interchangeable,
- 3PE derived strain has a shorter analysis time in respect to other techniques,
- 4D analysis of longitudinal deformation leads to a significant underestimation of longitudinal strain,
- a higher number of segments are not analyzable with 4DE,
- agreement between 2DE and 3PE for longitudinal strain is better than 2DE and 4DE,
- differences in longitudinal strain values do not appear to be influenced by differences in volume rate/frame rate acquisition.

Our data regarding the feasibility of four- and two-dimensional evaluation of longitudinal strain are in agreement with previous studies published in the literature [9, 10] that have shown a high feasibility for these techniques. Few studies however aimed to assess the feasibility of triplane echocardiography in the assessment of longitudinal deformation by means of speckle tracking. We reported a feasibility of 3PE-derived strain of 88.1%, which is higher with respect to that recently reported by Negishi et al. of only 47% [11]. We also identified the apical long-axis view as the major source of bias in comparison with 2D strain. 

In this study, we were able to identify that 4DE has longer times for analysis with respect to other techniques and that 3PE analysis shows the shortest times for analysis. Our data are partially in contrast with previous studies [12] that identified shorter averaged times for the acquisition (not assessed in the present work) and analysis of 4DE. During analysis, manual adjustment of the ROI was more frequently needed with 4DE in particular for the correct delineation of the epicardial border. Frequent manual adjustment with 4DE, coupled with a generally longer time for software elaboration of speckle pattern inside the ROI, could lead to a significant increase in the time spent for analysis. It has to be noted that preliminary steps required for the analysis of 4D strain allow the assessment of parameters of ventricular function including volumes, ejection fraction, LV mass, and sphericity index that should require several additional measurements with the other techniques. 

Our results about segmental feasibility showed that 4DE is characterized by a significant lower number of segments that could be satisfactorily included in the analysis. We have also shown that segments excluded have a regional pattern inside the 17-segment model of the LV, with basal and apical segments frequently excluded from the analysis. These data are again in partial contrast with previous findings by Pérez de Isla [9] that identified a higher number of analyzable segments using 3D speckle tracking (72.4%) compared with 2D speckle tracking (52.0%) in the analysis of longitudinal and radial (not assessed in this study) strains.

A significant underestimation of longitudinal strain with 4DE has been found in our study. Our findings are in agreement with previous studies showing a preeminent underestimation of longitudinal strain with 4D echo [11, 12] but in contrast with findings by Pérez de Isla [9] that showed similar values in the assessment of LV longitudinal strain as compared with 2D echo. We consider that differences in strain values between techniques are probably consequences of the speckle pattern inside the ROI that differ between 4DE and 2DE or 3PE. Bidimensional speckle pattern inside the ROI loses in fact the *z*-direction of the space [13]; moreover, speckle drop back from the ROI and foreshortened views, a potential source of errors with bidimensional analysis, could generate differences in tracking between the techniques. Though hypothesis generating, we also believe that segmental exclusion with 4DE (even if always inferior to 4 segments) could represent a potential source of bias. In fact, segments more frequently excluded from the analysis also included the apical region and the apex that physiologically show the highest strain values [14]. 

We also demonstrated that mean bias between techniques is similar in healthy subjects and in patients with pathological conditions potentially influencing longitudinal strain. This finding is consistent with the fact that differences between strain values are intrinsic to the specific technique used for analysis rather on patients' characteristics and underlines the need for technical specific reference values.

In addition, we were not able to demonstrate any influence of temporal resolution as a source of bias between the techniques as shown by the non-significant relationship between differences in frame rate/volume rate and the corresponding differences in strain values. Moreover, when patients were subdivided according to a higher (>40 volumes/sec) or lower volume rate acquisition, no differences in agreement and feasibility were observed. In authors' opinion, increasing volume rate acquisition is not necessarily related to better agreement and feasibility; there is in fact a trade-off between temporal and spatial resolution and increasing volume rate above a minimum required level that could be not superior for strain analysis. This has been defined as the frame rate paradox [11].

Differences among studies could be also a consequence of different software used for the analysis. Intervendor variability [15, 16] appears in fact to be an important limitation of 4DE in particular for speckle tracking analysis and could generate spurious findings in the literature. This is an important limitation for 4D analysis of myocardial deformation because it leads to disagreement in the literature and undermines the reliability of this new method for the assessment of myocardial function.

In conclusion, the current study yielded new lights into the developing field of speckle tracking echocardiography by systematically exploring the feasibility of different methods in a clinical scenario. We explored for the first time segmental feasibility and time spent for analysis and we highlighted differences actually displayed by each technique. We hope that these findings could be useful to standardize and critically evaluate not only the potentialities but also the actual limitations of speckle tracking imaging in daily clinical practice. 

## 5. Limitations

The present study did not explore feasibility, agreement, and reproducibility between radial and circumferential strain values among 2DE and 4DE (3PE acquisition allows in fact the assessment of only longitudinal strain). Such components of myocardial deformation are important for a global and complete evaluation of myocardial performance. In addition, a test-retest analysis was not included in the study's design. Finally, the finding of a nonconsiderable influence of temporal resolution upon intertechnical biases is an indirect observation, and further studies specifically addressing this issue are needed.

## 6. Conclusions

Real time multidimensional echocardiographic techniques for the assessment of longitudinal deformation are feasible but show a fewer number of segments considered as appropriate for analysis. 3PE derived strain has a good relationship with 2D strain, while 4DE significantly underestimates LV longitudinal deformation. Echocardiographic techniques for the assessment of longitudinal deformation are therefore not interchangeable, and further studies are needed to assess specific reference values.

## Figures and Tables

**Table 1:** Clinical characteristics and echocardiographic parameters of the study population.

	Values	Range
Age (years)	41.7 ± 16.5	14–76
Sex (M/F)	42/59	—
PFO closure, *n* (%)	19 (18.8)	—
ASD closure, *n* (%)	16 (15.8)	—
Arterial hypertension, *n* (%)	24 (23.8)	—
Athletes, *n* (%)	23 (22.8)	—
Healthy subjects, *n* (%)	19 (18.8)	—
BSA (m^2^)	1.84 ± 0.20	1.38–2.35
BMI (kg/m^2^)	25.5 ± 3.9	18.6–36.1
Heart rate (beats/min)	71.6 ± 12.6	46–109
Systolic blood pressure (mmHg)	124.6 ± 18.7	80–180
Diastolic blood pressure (mmHg)	77.0 ± 9.7	55–105
LV-EDV (mL)	97.6 ± 27.5	43–221
LV-ESV (mL)	42.6 ± 15.9	15–106
LV-SV (mL)	55.0 ± 14.2	28–115
LV-EF (%)	57.0 ± 6.7	34–76
LV-M (g)	130.4 ± 21.8	76–196
LV-CO (L/min)	3.91 ± 1.14	1.7–7.0
LV-SpI	0.38 ± 0.09	0.19–0.58

Data are expressed as number of subjects (*n*) and percentages (%) or mean ± standard deviation. PFO: patent foramen ovale; ASD: atrial septal defect; BSA: body surface area; BMI: body mass index; LV: left ventricle; LV-M: left ventricular mass; EDV: end diastolic volume; ESV: end systolic volume; SV: stroke volume; EF: ejection fraction; CO: Cardiac Output; SpI: sphericity index.

**Table 2:** Reproducibility analysis.

	Intraobserver		Interobserver	
	COV (%)	ICC (95% CI)	COV (%)	ICC (95% CI)
2D Strain (%)	3.5 ± 3.2	0.96 (0.89–0.98)	5.9 ± 4.9	0.95 (0.88–0.97)
3P Strain (%)	4.2 ± 3.7	0.95 (0.87–0.98)	7.8 ± 3.7	0.94 (0.78–0.97)
4D Strain (%)	7.8 ± 6.0	0.96 (0.90–0.98)	10.9 ± 6.5	0.92 (0.88–0.95)
Apical views				
2D Strain (%)				
4 chamber view	6.5 ± 6.3	0.91 (0.78–0.97)	8.8 ± 8.4	0.88 (0.77–0.90)
2 chamber view	5.6 ± 4.9	0.92 (0.81–0.97)	7.5 ± 6.7	0.87 (0.75–0.96)
Apical long axis view	6.3 ± 5.4	0.88 (0.68–0.95)	9.8 ± 7.6	0.84 (0.91–0.99)
3P Strain (%)				
4 chamber view	7.1 ± 5.8	0.87 (0.68–0.95)	9.5 ± 4.7	0.84 (0.67–0.94)
2 chamber view	7.8 ± 5.2	0.89 (0.71–0.96)	9.9 ± 7.3	0.87 (0.69–0.96)
Apical long axis view	6.2 ± 6.3	0.83 (0.83–0.97)	8.4 ± 7.6	0.80 (0.75–0.84)

COV: coefficient of variability; ICC: intraclass correlation coefficient. COV is expressed as mean ± SD.
